# Synthesis of MWCNTs by chemical vapor deposition of methane using FeMo/MgO catalyst: role of hydrogen and kinetic study

**DOI:** 10.1038/s41598-023-48456-z

**Published:** 2023-11-29

**Authors:** Chawalkul Chotmunkhongsin, Sakhon Ratchahat, Weerawut Chaiwat, Tawatchai Charinpanitkul, Apinan Soottitantawat

**Affiliations:** 1 Center of Excellence in Particle and Material Processing Technology, Department of Chemical Engineering, Faculty of Engineering, Chulalongkorn University, Bangkok, 10330 Thailand; 2 Department of Chemical Engineering, Faculty of Engineering, Mahidol University, Nakhon Pathom, 73170 Thailand

**Keywords:** Catalysis, Chemical engineering, Materials chemistry, Chemical synthesis, Chemical engineering, Carbon nanotubes and fullerenes, Synthesis and processing, Two-dimensional materials, Carbon capture and storage, Chemistry, Nanoscience and technology

## Abstract

This study aims to investigate the role of hydrogen on CNTs synthesis and kinetics of CNTs formation. The CNTs were synthesized by catalytic chemical vapor deposition of methane over FeMo/MgO catalyst. The experimental results revealed that hydrogen plays an important role in the structural changes of catalyst during the pre-reduction process. The catalyst structure fully transformed into metallic FeMo phases, resulting in an increased yield of 5 folds higher than those of the non-reduced catalyst. However, the slightly larger diameter and lower crystallinity ratio of CNTs was obtained. The hydrogen co-feeding during the synthesis can slightly increase the CNTs yield. After achieving the optimum amount of hydrogen addition, further increase in hydrogen would inhibit the methane decomposition, resulting in lower product yield. The hydrogenation of carbon to methane was proceeded in hydrogen co-feed process. However, the hydrogenation was non-selective to allotropes of carbon. Therefore, the addition of hydrogen would not benefit neither maintaining the catalyst stability nor improving the crystallinity of the CNT products. The kinetic model of CNTs formation, derived from the two types of active site of dissociative adsorption of methane, corresponded well to the experimental results. The rate of CNTs formation greatly increases with the partial pressure of methane but decreases when saturation is exceeded. The activation energy was found to be 13.22 kJ mol^−1^, showing the rate controlling step to be in the process of mass transfer.

## Introduction

Carbon nanotubes (CNTs) were discovered over three decades ago in 1991^1^. The unique properties of CNTs have been extensively researched and applied in several applications such as semi-conductors, batteries, reinforced materials and universal composite materials^2^. The synthesis of CNTs can be simply achieved by a transformation of carbon-containing substances under suitable conditions to carbon atoms, recombination of six-member ring graphene structure. The catalytic decomposition of hydrocarbons such as methane can produce CNTs grown on the catalyst surface, while hydrogen (H_2_) as gas product is produced^3–6^. From above, H_2_ gas generally found during CNTs synthesis. There are many researched show that all of these process success to produce a CNTs, which are H_2_ free process^7^, H_2_ was used only to pre-reduction of catalyst process but not to CNTs formation process^8^, or H_2_ was used in the entire process from reduction of catalyst to synthesis of CNTs process^9,10^ as shown in Table 1.

**Table 1:** Condition for synthesis of CNTs.

Catalyst	Feedstock	Reduction	Product	Ref.
MetalSO_4_/SiO_2_ (Fe, Ni, Co)	CO/Ar	No	SWCNTs	Wang^7^
CoMo/MgO	CH_4_/Ar/H_2_O	No	SWCNTs	Alijani^30^
Ferrocene + nickelocene	Ar	No	SWCNTs	Chiang^31^
Fe, Ni, Co/MgO, SiO_2_, Al_2_O_3_	CH_4_/N_2_	No	SW/MWCNTs	Liu^32^
Ferrocene + sulfur	CH_4_/Ar–H_2_	No	SWCNTs	Yadav^33^
CoMo/MgO	C_2_H_4_/N_2_–H_2_	No	MWCNTs	Chang^34^
Fe/MgO	CH_4_/He	Yes	SWCNTs	Abdullahi^8^
CoSO_4_/SiO_2_	CO	Yes	SWCNTs	Wang^35^
CoMo/quartz coating	EtOH/Ar–H_2_	Yes	SW/DWCNTs	Inoue^10^
FeMo/SiO_2_	C_2_H_4_/N_2_–H_2_	Yes	MWCNTs	Chang^9^

The roles of H_2_ on CNTs synthesis has been extensively studied. Piedigrosso et al.^11^ reported that addition of H_2_ would reduce carbon yield by elimination of both CNTs and amorphous carbon through hydrogenation. In contrast, some research works found that the co-feeding of H_2_ during CNTs synthesis can increase CNT yield^12–18^ by selective elimination of amorphous carbon. The other effects of H_2_ were widely reported; the H_2_ concentration made different CNTs diameter^14,15,18^, included effect to formation of SWCNTs some researcher found H_2_ hinder formation of SWCNTs^19,20^. On the other hand, the H_2_-free or less concentration of H_2_ process could not synthesis a SWCNTs^21^, including to MWCNTs^22^. The reason of this effect was reported in the same way that is H_2_ has a role to control the size, phase, and morphology of catalysts. For CNTs synthesis, catalyst is the one necessary factor to control CNTs formation, for example the same Fe-based catalyst but one is α-Fe phase and other one is Fe_3_C phase, the formation of CNTs occur with a huge different in yield, diameter, and CNT characteristics. He et al.^23^ and Torres et al.^6^ reported Fe_3_C phase resulted in the formation of bamboo-like CNTs, additionally Fe_3_C forming smaller diameter CNTs but less yield than α-Fe^12,24^.

Research has shown that the addition of Mo can increase the efficiency of CNTs synthesis by increasing yield and enhancing better stability at high temperatures^8,25^. Furthermore, mechanisms of the effects of hydrogen on the synthesis of CNTs using FeMo/MgO as a catalyst have not been reported.

Recently, the CNTs manufactures have been developed largely that directly affecting to the continued decline in sale prices and expected that the price will further go down globally^26^, even so CNTs are still expensive, approximately price of SWCNTs and MWCNTs are 38,000฿^27^ and 3400฿^28^ per gram, respectively. The market for MWCNTs has a decline in large-scale production; however, there still remains global demand of > 2000–2500 tons per annum with increased demand in composites, automotive and aerospace applications and especially as battery additives in Asia^29^. In contrast, H_2_ demand is increasing consistently. Therefore, to compete in the CNTs market, manufacturers should balance costs to suit the CNTs properties.

This work focuses on studying the role of H_2_ to whole process of CNTs synthesis by using FeMo/MgO as a catalyst and finding out in-depth behavior of each process to guide further improvements in CNTs manufacturing process as well as its reaction kinetic.

## Experimental

### Catalyst preparation

**Figure 1:**
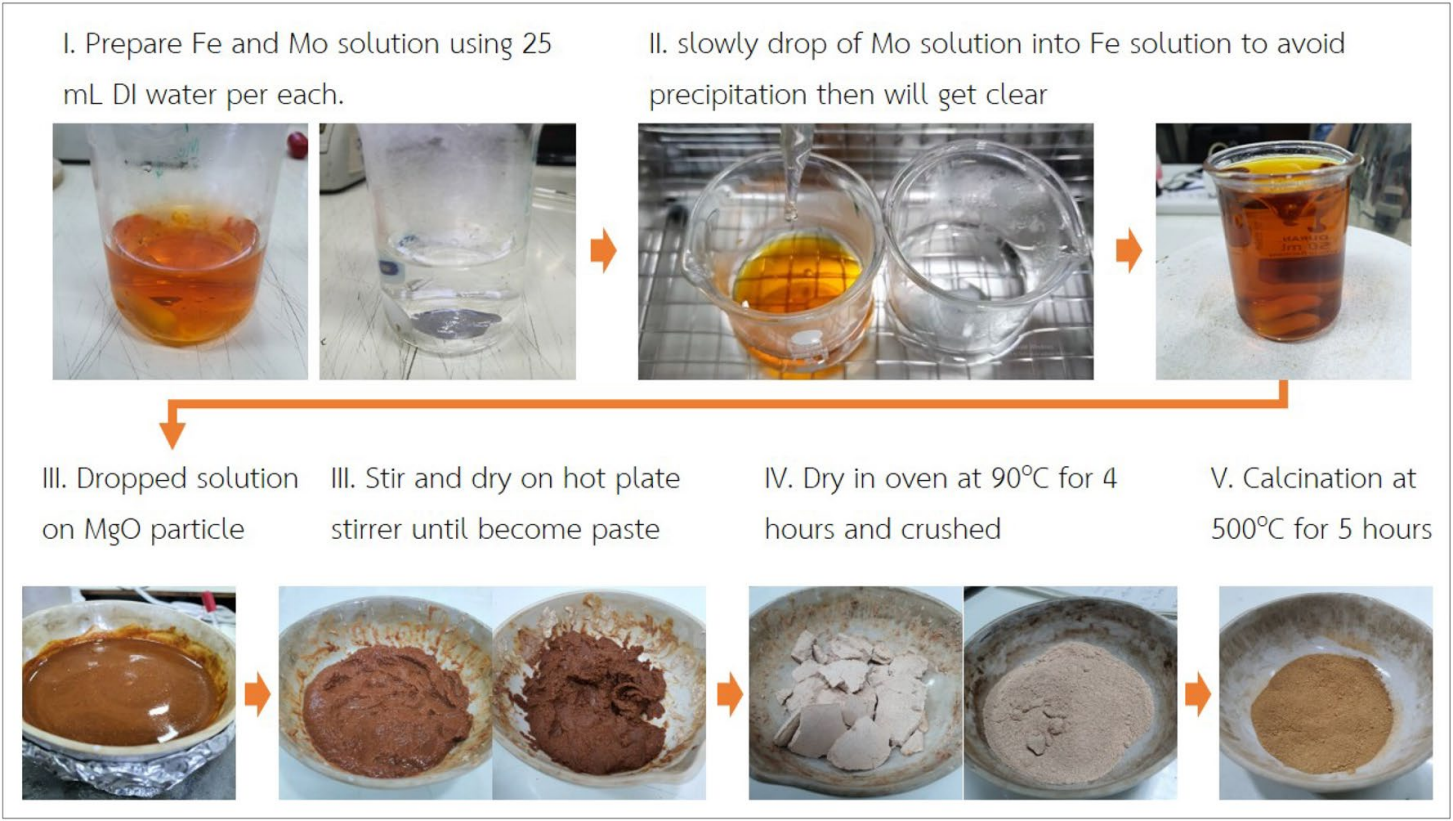
Preparation of FeMo/MgO by impregnation method.

FeMo/MgO catalyst with 30%wt metals loading was prepared by impregnation method. The iron (III) nitrate nonahydrate (Fe(NO_3_)_3_.9H_2_O, 98%, AR, Loba chem.) and ammonium heptamolybdate ((NH_4_)_6_Mo_7_O_24_, AR, KEMAUS ) solution were prepared as precursors of Fe and Mo with mass ratio of Fe:Mo equal to 2:1, then slowly drop of Mo solution into Fe solution to avoid precipitation. The FeMo solution was dropped on magnesium oxide fine particle (MgO, pharma, Applichem Panreac.). The mixture was stirred and dried on hot plate stirrer at 90 °C until forming of paste, then dried in oven with the same temperature for 4 h and calcination at 500 °C for 3 h. The FeMo/MgO in oxide form is ready to use for the reaction. The images of the experimental process are shown in Fig. 1.

### CNTs synthesis

**Figure 2:**
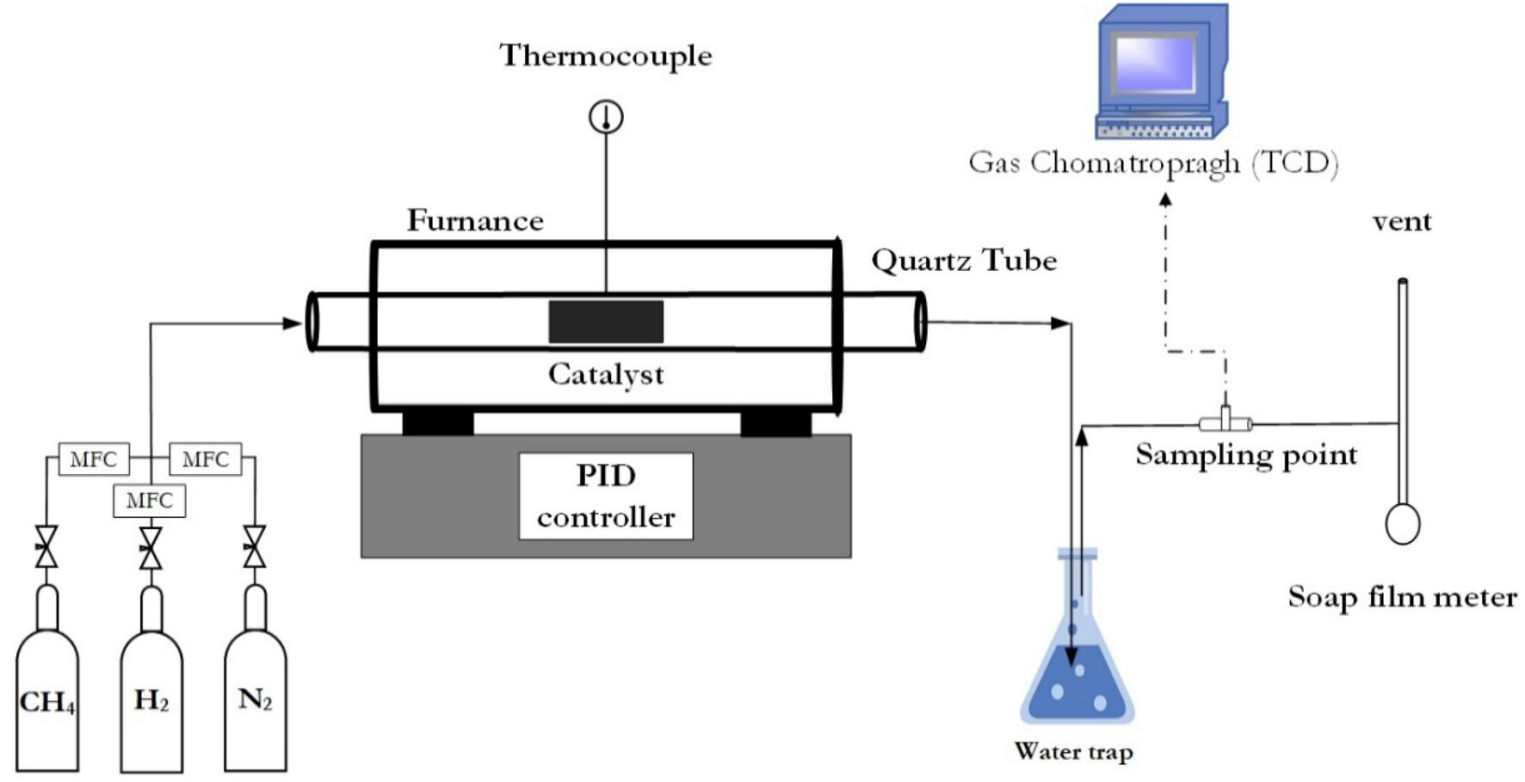
Schematic diagram of CCVD method.

CNTs were synthesized by the catalytic chemical vapor deposition (CCVD) method using methane as a carbon source. The schematic diagram was shown in Fig. 2. Firstly, 0.5 g of FeMo/MgO catalyst was put in a quartz boat, then placed in the quartz tube reactor center. This CCVD process started with N_2_ fed into the reactor and heated up from room temperature until it reached desired temperature by linearly increasing at a rate of 10 °C min^−1^.

The total gas flow rate was controlled with following each condition, which calibrated by soap film meter. The effluent gas was collected and analyzed with Gas Chromatography (GC-TCD 8A, Shimadzu). Conditions for GC analysis were set as follows (3 mm ∅ × 2 m column, INJ/DET: 120 °C, COL: 100 °C, active carbon packing: 0.2–0.25 mm, and He carrier: 40 ml min^−1^). Until the end of the reaction (180 min), the system was cooled to room temperature in N_2_ gas flow. The CNTs were formed at the surface of the catalyst in the quartz boat.

The catalytic performance in terms of CH_4_ conversion, Carbon yield, g-CNTs/g-catalysts, and calculated H_2_ flow rate were calculated according to Eqs. (1)–(4), respectively.

1 $$X_{{{\text{CH}}_{4} }} \left( \% \right) = \frac{{\left[ {{\text{CH}}_{4} } \right]_{in} \times F_{in} - \left[ {{\text{CH}}_{4} } \right]_{out} \times F_{out} }}{{\left[ {{\text{CH}}_{4} } \right]_{in} \times F_{in} }} \times 100$$

2 $$Carbon \;yield \left( \% \right) = {}\frac{product \;weight \left( g \right) - catalyst \;weight \left( g \right)}{{carbon \;feed \left( g \right)}}\times100 \%$$

3 $$\frac{g - CNTs}{{g - catalysts}} = {}\frac{product \left( g \right) - catalyst \;weight \left( g \right)}{{catalyst \;weight \left( g \right)}}$$

4 $$Calculated\;H_{2} \;flow\;rate\left( {{\text{ml}}/{\text{min}}} \right) = F_{outlet} {-}F_{N2} {-}F_{{unreacted,\;{\text{CH}}_{4} }}$$

The kinetics investigation in “Kinetic study of CNTs synthesis” section will perform experiment with H_2_ pre-reduction process (R-woH_2_). The product yield used to calculate yield rate as an Eq. (5). The reduced catalyst weight after the reduction process will be 0.75 times to the initial catalyst weight. Rate of reaction will be calculated by Eq. (6)

5 $$Product\;yield\left( g \right) = g_{product} - g_{reduced - catalyst} ;g_{reduced - catalyst} = 0.75xg_{catalyst}$$

6 $$r_{CNTs} \left( {gg_{cat.}^{ - 1} s^{ - 1} } \right) = \frac{product\;yield \left( g \right)}{{ g_{reduced - catalyst} \times reaction\;time \left( S \right)}}$$

### Characterization

The study of morphology, internal, and external structure was examined using imaging process. The external structure of as-prepared and purified product was observed by Field Emission Scanning Electron Microscopes (FE-SEM, HITACHI SU-8010). The internal structure, type of CNTs, number of walled, CNTs growth mechanism (Tip, based growth), CNTs and catalyst diameter size were observed by Transmission Electron Microscopes (TEM, JEOL JEM-2100 Plus). Sample preparation for TEM analyzed was prepared by following step; the product was dispersed in ethanol (99.5v/v%, Sigma-Aldrich) and then dropped onto a 300 mesh of copper grid coated with carbon film. As-prepared CNTs and catalysts diameter size were measured from SEM, and TEM images by using ImageJ software. Crystallinity and structure of CNTs and catalysts were analyzed for study of changing in the sample, because of the controlled processes. X-ray diffraction (XRD) with the angle range scan 5°–80° and TEM selected area electron diffraction (SAED) were used to specify crystallinity, phase change, and crystal size. The crystallinity of the synthesized carbon products, which validated with qualitative analysis of the proportion between crystalline carbon and amorphous carbon was carried out using 532 nm laser light source Raman spectroscopy (Raman NT-MDT model: NTEGR-SPECTRA). The purity of as-prepared product was measured from the thermo-gravimetric analysis (TGA), by observing the weight loss in expected temperature range.

## Results and discussion

### Roles of hydrogen

#### Effect of the presence of H_2_

In the study roles of H_2_, there are two variables that need to be studied which are, the pre-reduction of catalyst and the H_2_ feeding during CNTs formation. Four processes: H_2_ free process (no pre-reduction process and without H_2_ feed during CNTs formation, nR-woH_2_), H_2_ co-feed process (no pre-reduced process and with H_2_ feed during CNTs formation, nR-wH_2_), H_2_ pre-reduction process (pre-reduction process and without H_2_ feed during CNTs formation, R-woH_2_), and H_2_ combined process (pre-reduction process with H_2_ feed during CNTs formation, R-wH_2_) which concluded in Table 2, were used to investigate H_2_ roles through catalytic activity and product properties with following results.

**Table 2:** Experimental conditions.

Process	Reduction 30–900 °C 40 min (150 ml min^−1^)	Reaction 900 °C 180 min (200 ml min^−1^)
H_2_ free process (nR-woH_2_)	N_2_ = 150	CH_4_:N_2_ = 50:150
H_2_ co-feed process (nR-wH_2_)	N_2_ = 150	CH_4_:N_2_:H_2_ = 50:50:100
H_2_ pre-reduction process (R-woH_2_)	N_2_:H_2_ = 50:100	CH_4_:N_2_ = 50:150
H_2_ combined process (R-wH_2_)	N_2_:H_2_ = 50:100	CH_4_:N_2_:H_2_ = 50:50:100

**Figure 3:**
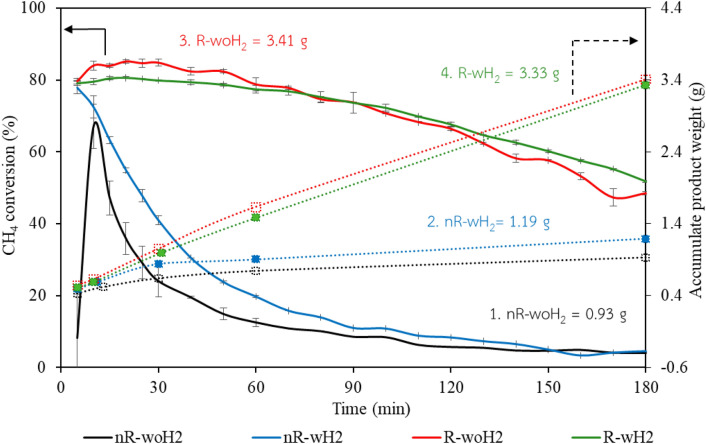
Catalytic activity through (solid line) methane conversion and (dotted line) product weight.

The catalytic activity was examined over CCVD for synthesis of CNTs process. From the experiment, FeMo/MgO catalyst is capable of converting CH_4_ in all processes as shown by methane conversion in Fig. 3.

**Figure 4:**
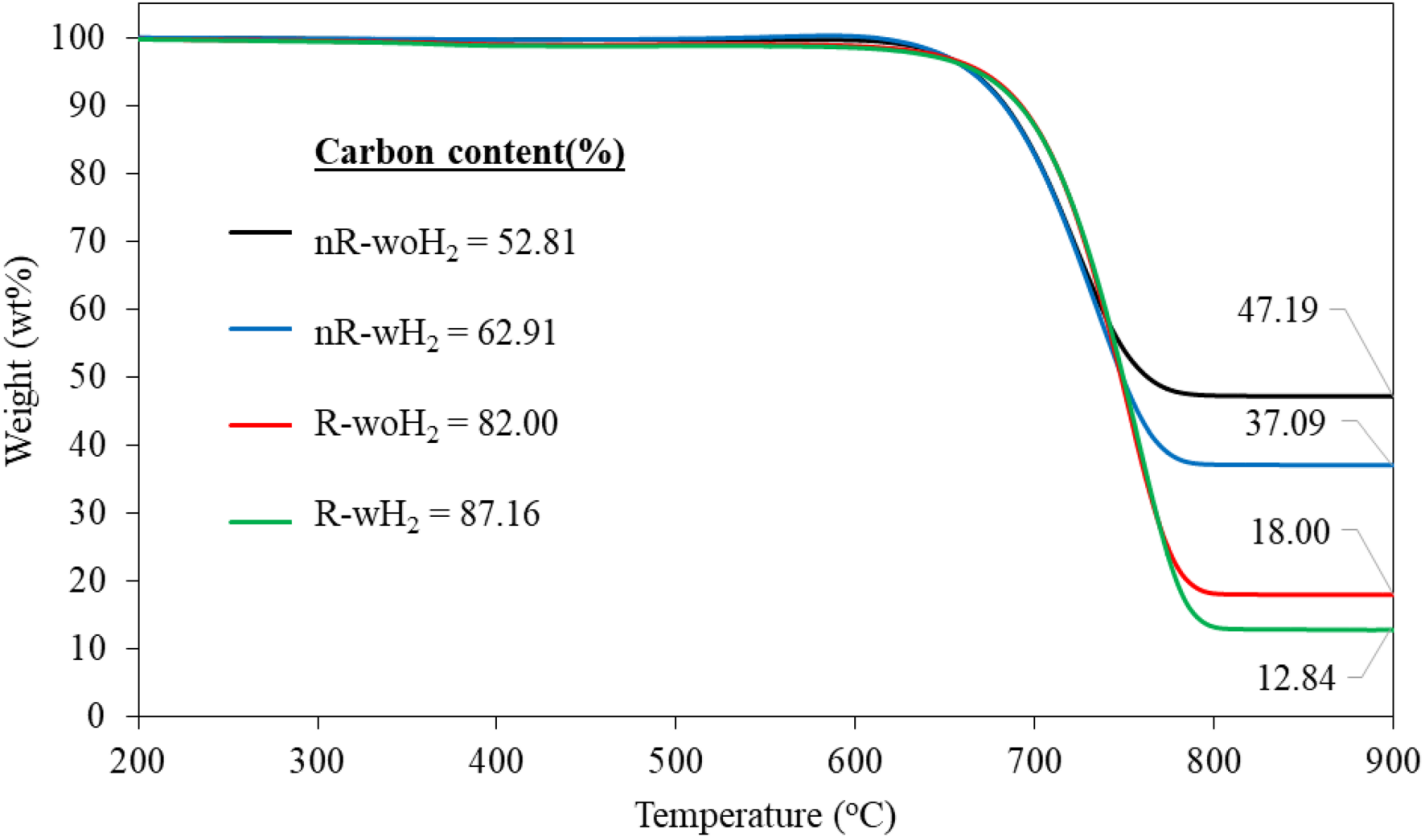
TGA of as-prepared CNTs by comparing each H_2_ processes.

Methane conversions decreased over time because of catalyst deactivation. The formation of product CNTs obstructed the active surface of catalysts. The catalyst performance depends on methane conversion, deactivated rate, and quantity of final product. It was found that R-woH_2_ was the most effective process with a 3.41 g final product followed with R-wH_2_ (3.33 g), nR-wH_2_ (1.19 g), and nR-woH_2_ (0.93 g), respectively. The thermal stability of the product was observed with thermo-gravimetric analysis (TGA) in air atmosphere, as shown in Fig. 4.

From Fig. 4, it was found that product begins to decompose at temperatures of 600 °C and above. Decomposition curves showed similar decomposed characteristics or referring to the same group of products. Residuals from decomposition under air atmosphere are catalyst and supported materials which are highly thermal stability than carbon product, therefore we can get carbon content from TGA. The percentage carbon content corresponds to the yield. Products from pre-reduction of catalyst process (R-woH_2_, R-wH_2_) have higher carbon content than non-reduced processes.

**Figure 5:**
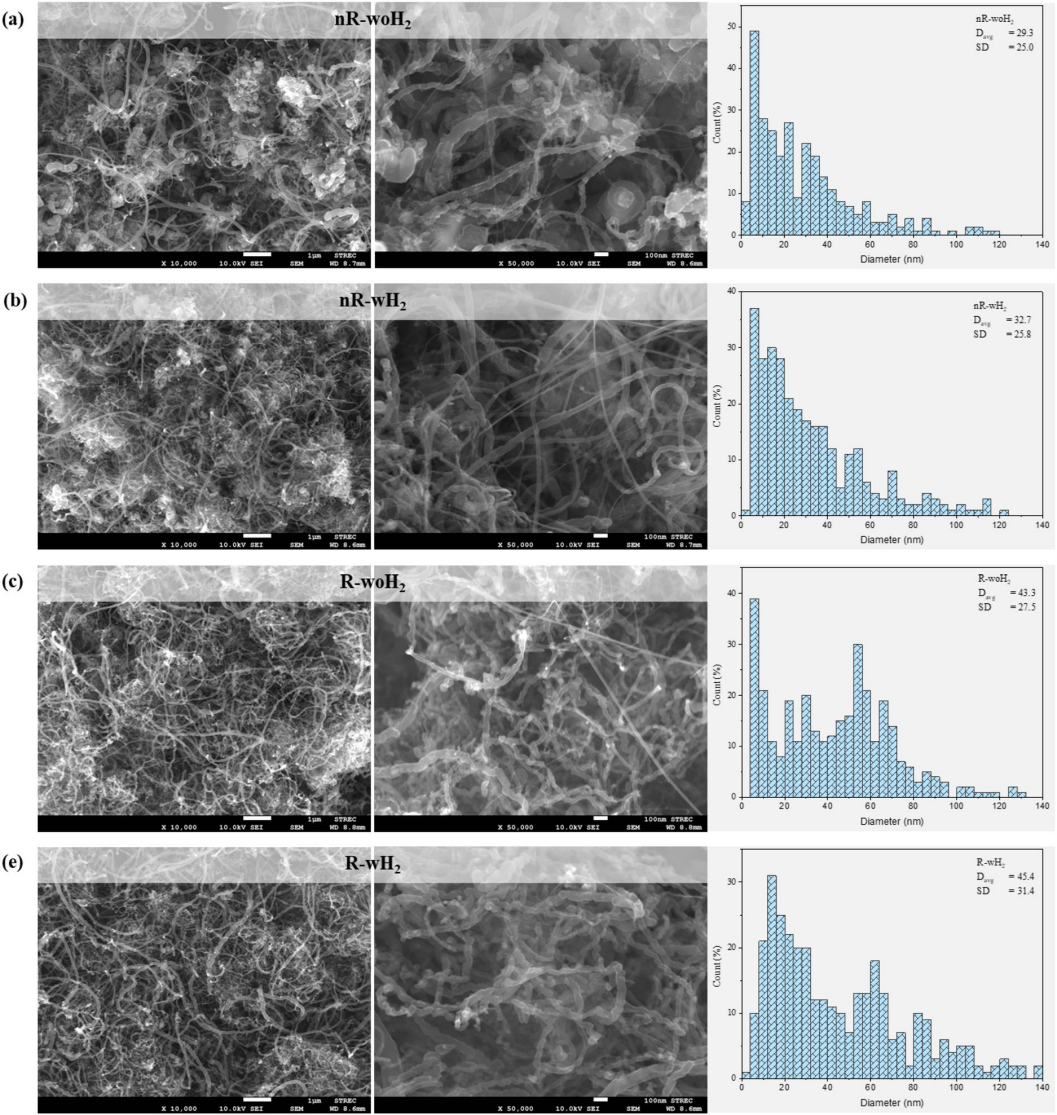
SEM images of as-prepared CNTs with magnification ×10,000, ×50,000, and CNTs diameter size distribution from (**a**) nR-woH_2_, (**b**) nR-wH_2_, (**c**) R-woH_2_, and (**d**) R-wH_2_, respectively.

The morphology of as-prepared products from various processes was investigated by image processing through scanning electron microscope (SEM) as shown in Fig. 5. Dense CNTs were found in all SEM images included with some amorphous carbon. Considering the CNTs products, the diameter of MWCNTs was measured from over 200 samples at 10,000×. magnification combined with over 100 samples at 50,000×. magnification by using ImageJ software to produce a diameter size distribution of as-prepared CNTs from each process.

The diameter size distribution of CNTs represents non-uniform product, ranging from tiny CNTs (smaller than 5 nm) to huge CNTs (larger than 100 nm), as a result of improper catalyst preparation method, thus affecting the active size which is expressed as the diameter of CNTs^36^*.* Considering the mean diameter, CNTs from non-reduced processes: nR-woH_2_ and nR-wH_2_ were approximately the same sizes (29.29 and 32.72 nm) meanwhile, the reduced process: R-woH_2_ and R-wH_2_ have similar in diameter (43.25 and 45.41 nm) but were larger than CNTs-nR processes.

**Figure 6:**
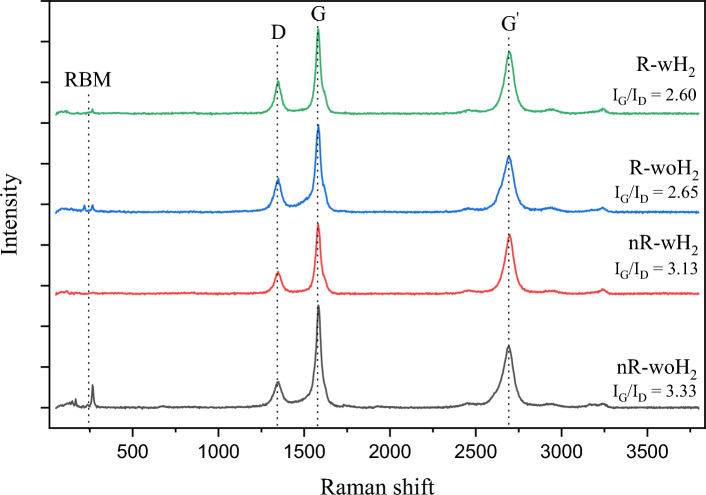
The representive Raman shifts of CNTs prepared by different conditions.

Qualitative analysis of the proportion between crystalline carbon and amorphous carbon was carried out using 532 nm laser light source Raman spectroscopy. Raman is used to determine proportions between graphitize carbon and disorder or amorphous carbon by the relative height of G band (~ 1580 cm^−1^) and D band (~ 1350 cm^−1^) that represent graphitic and disorder carbon, respectively. The G/D peak intensity ratios (I_G_/I_D_) of a carbonaceous product were investigated more than 5 points for each process to confirm the results of the analysis and prevent false sampling. Examples of the Raman analysis result are shown in Fig. 6. Average I_G_/I_D_ ratio and other detailed data for the study of overview H_2_ roles are shown in Table 3.

**Table 3:** Summary data for the study of overview H_2_ roles.

Experimental	%Yield	g-CNTs/g-catalysts	%C (TGA)	D_avg_ (nm)	Avg. I_G_/I_D_
nR-woH_2_	10.16	0.85	52.81	29.28 ± 25.03	3.14 ± 0.61
nR-wH_2_	10.85	1.37	62.91	32.72 ± 25.80	3.07 ± 0.49
R-woH_2_	67.77	5.77	82.00	43.25 ± 27.53	2.58 ± 0.49
R-wH_2_	64.88	5.61	87.16	45.41 ± 27.53	2.56 ± 0.24

From the Raman analysis, it was found that the products formed by non-reduced processes have higher I_G_/I_D_ ratio than reduced processes which approximately are 3.1 and 2.5, respectively. Observed that the addition of H_2_ during the formation of CNTs did not cause a difference in the crystallinity ratio (more detailed in “Hydrogenation reaction” section). Therefore, there was a possibility that the crystallinity ratio was also controlled by the catalyst structure.

In the study of H_2_ roles, the samples were clearly divided into two groups which are non-reduced processes and reduced process. Non-reduced process (nR-woH_2_ and nR-wH_2_) produces products with smaller diameters and higher crystallinity ratios than reduced processes (R-woH_2_ and R-wH_2_), but reduced processes have a higher yield than 5 times (~ 1 to ~ 5) of non-reduced process.

The structure of the catalyst and as-prepared product in each process were investigated by XRD method to describe the mechanisms that cause changes in CNTs. Firstly, the H_2_-temperature programmed reduction (H_2_-TPR) analysis was used to investigate H_2_ consumption during temperature changes, which is a similar experimental to pre-reduction of catalyst process. In the reduced process, it was confirmed by H_2_-TPR that pre-reduction of catalyst from room temperature to 900 °C would allow the catalyst to remain structurally stable or without structural changes in the presence of hydrogen in the system^3^, as shown in Figure S1, that H_2_ is not consumed at 900 °C and above.

**Figure 7:**
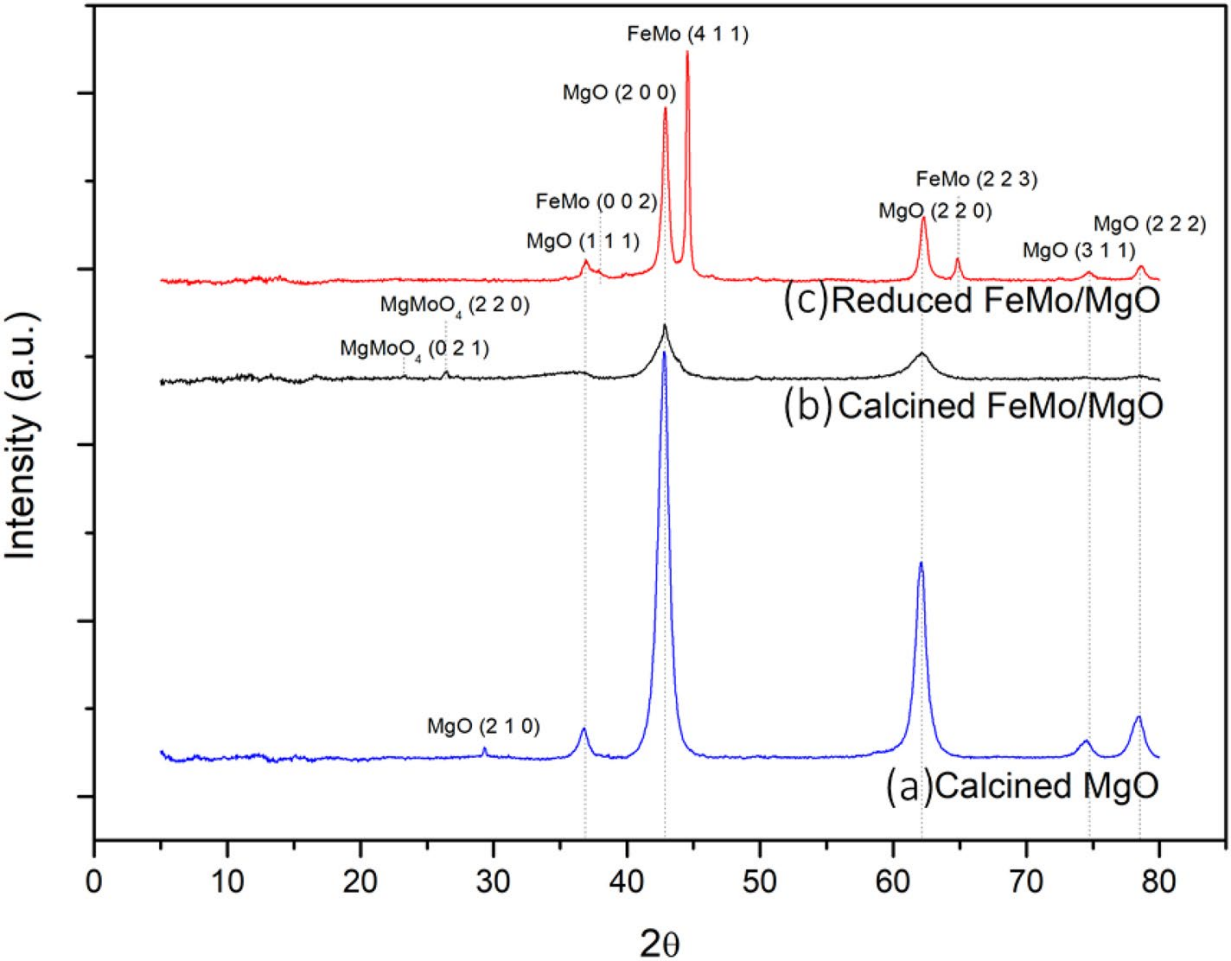
XRD patterns of (**a**) calcined MgO (**b**) calicned FeMo/MgO, (**c**) reduced FeMo/MgO.

XRD patterns of catalysts consisted of calcined MgO, calcined FeMo/MgO, and reduced FeMo/MgO are shown in Fig. 7. The catalyst phases were identified by the diffraction peaks. The diffraction peaks of magnesium oxide (MgO, PDF 01-071-1176) were observed in Fig. 7a which were cubic structures. Figure 7b exhibited the diffraction peaks of MgO impregnated with iron (Fe) and molybdenum (Mo), where only monoclinic MgMo_4_ at 23.2° (021), and 26.3° (220) ^37^ was found. Xu et al.^38^ reported an XRD analysis of calcined FeMo/MgO, revealing the structure of MgO, Fe_3_O_4_, and MgMo_4_. The results of the xrd diffraction were similarly blunt same as this work. XRD patterns of reduced FeMo/MgO was show in Fig. 7c, tetragonal FeMo exhibited the diffraction peak at 37.3° (002), 44.6° (411), and 64.7° (223)^39^.

Figure 7 found that the calcination process did not detect Fe-Mo interaction, FeMo was found after the catalytic reduction process. Therefore, the reduction of catalyst process changes the catalyst structure from the oxide form to metallic structure and establishes an interaction between iron and molybdenum.

**Figure 8:**
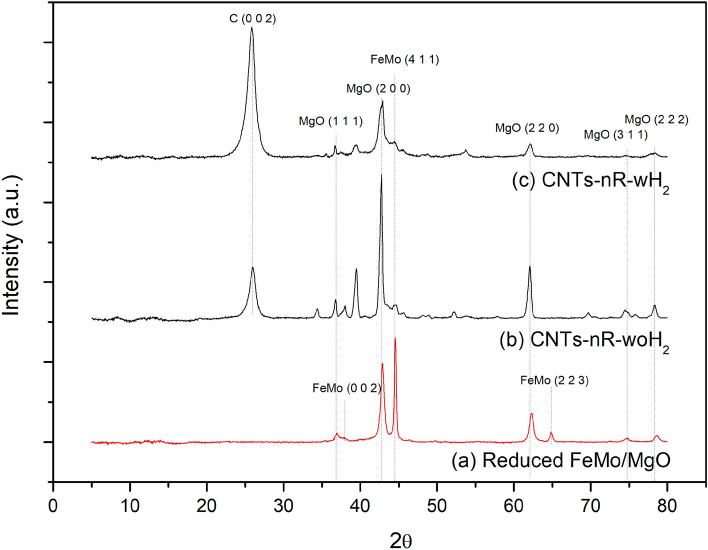
Comparing XRD patterns of (**a**) reduced FeMo/MgO (**b**) CNTs-nR-woH_2_, (**c**) CNTs-nR-wH_2_.

Comparison of product structure between reduced FeMo/MgO, non-reduced CNTs (nR-woH_2_), and reduced CNTs (R-woH_2_) is shown in Fig. 8. FeMo undetectable after CNTs growth from both of non-reduced and reduced processes, which may cause by carbon obscuring or active metal was encapsulated within CNTs. Diffraction patterns of hexagonal carbon or carbon nanotubes was found at 25.9° (002)^40^, which shown in Fig. 8b and c.

**Figure 9:**
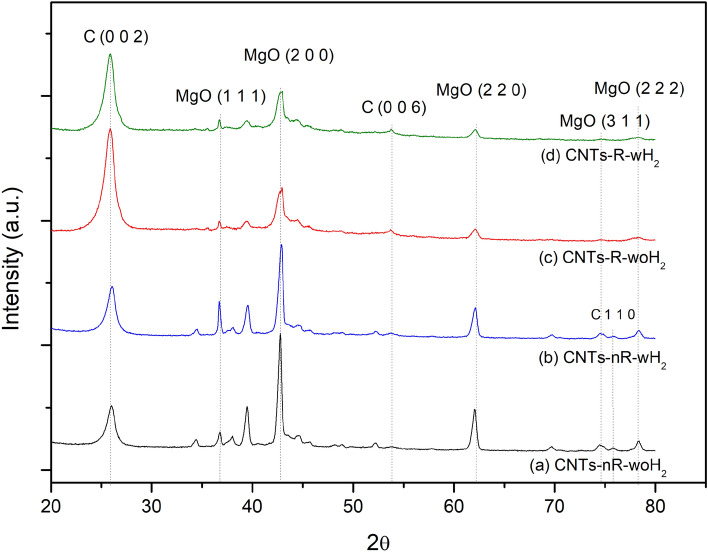
XRD patterns of as-prepared CNTs from (**a**) nR-woH_2_, (**b**) nR-wH_2_, (**c**) R-woH_2_, (**d**) R-wH_2_.

The XRD patterns of as-prepared CNTs are shown in Fig. 9. It was found that the product characteristics are divided into two groups in the same way as the properties of CNTs, which are non-reduced processes and reduced processes.

**Figure 10:**
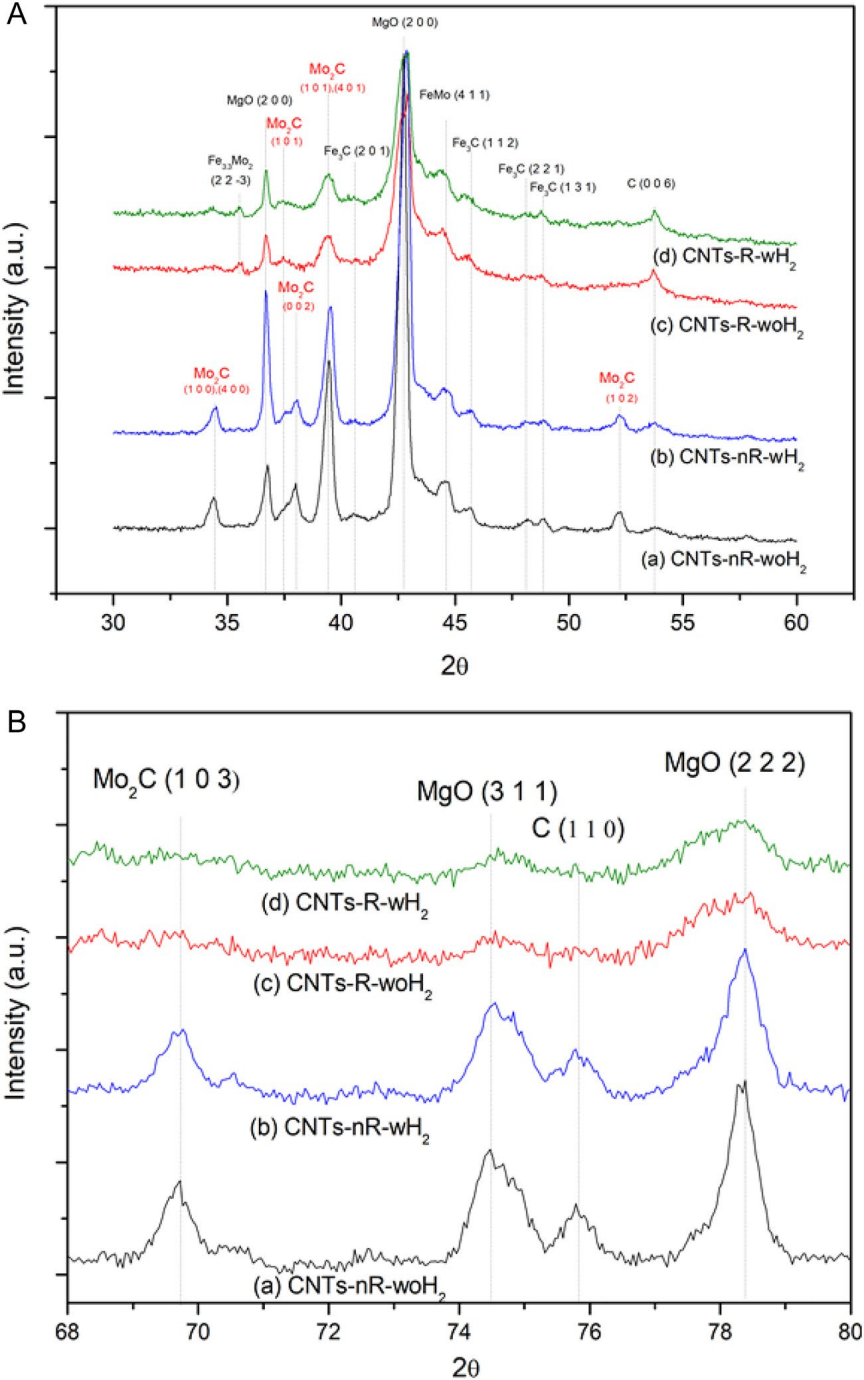
XRD patterns of as-prepared CNTs in ranges (**A**) 30°–60° (**B**) 68°–80° from (**a**) nR-woH_2_, (**b**) nR-wH_2_, (**c**) R-woH_2_, (**d**) R-wH_2_.

The details of the XRD analysis results in the 30°–60° and 68°–80° ranges are shown in Fig. 10A and B, respectively. The diffraction pattern exhibited iron carbide (Fe_3_C) that can be found in all processes. Meanwhile, diffraction peak of molybdenum carbide (Mo_2_C) is discovered in non-reduced processes. Therefore, in a non-reduced process, the catalyst is converted from oxide to carbide form. In addition, iron, and molybdenum interacted poorly, resulting in the formation of molybdenum carbide structures. These results show why synthesized CNTs by non-reduced and reduced processes differ.

Several previous studies have mentioned similar behaviors with Fe catalysts. He et al.^23^ reported that the carbide catalyst (Fe_3_C) will make CNTs with a smaller diameter but lower yield than the metallic catalyst (α-Fe). In this work, we found that non-reduced processes will be made the carbide catalyst, but reduced processes will be made metallic catalyst (FeMo). This is why products from the non-reduced and reduced processes are different.

The carbide formation of catalyst is described by Behr et al.^15^. They studied the H_2_-to-CH_4_ ratio in the feed gas. Carbide formation will occur when lower H_2_– to –CH_4_ ratio, while the metallic formation will occur when a higher H_2_-to-CH_4_ ratio. Reduced processes in this work occur in H_2_ and N_2_ atmosphere, in the meaning there is no carbon (CH_4_) in the system, that made a FeMo metallic, but for non-reduced processes, catalyst will be reduced by H_2_ gas dissociated from CH_4_, because CH_4_ can be decomposed by the oxide form of the catalyst^41^. The reaction product hydrogen gas may make the reduction form of the catalyst with presence of carbon atoms on catalyst surface that caused a carbide catalyst.

Therefore, from the study of H_2_ roles, it was found that the characteristics of CNTs, whether yield, diameter, and proportion of crystallinity, are determined by the structure of catalyst that was controlled by the pre-reduction process.

#### Effect of H_2_ concentration

**Figure 11:**
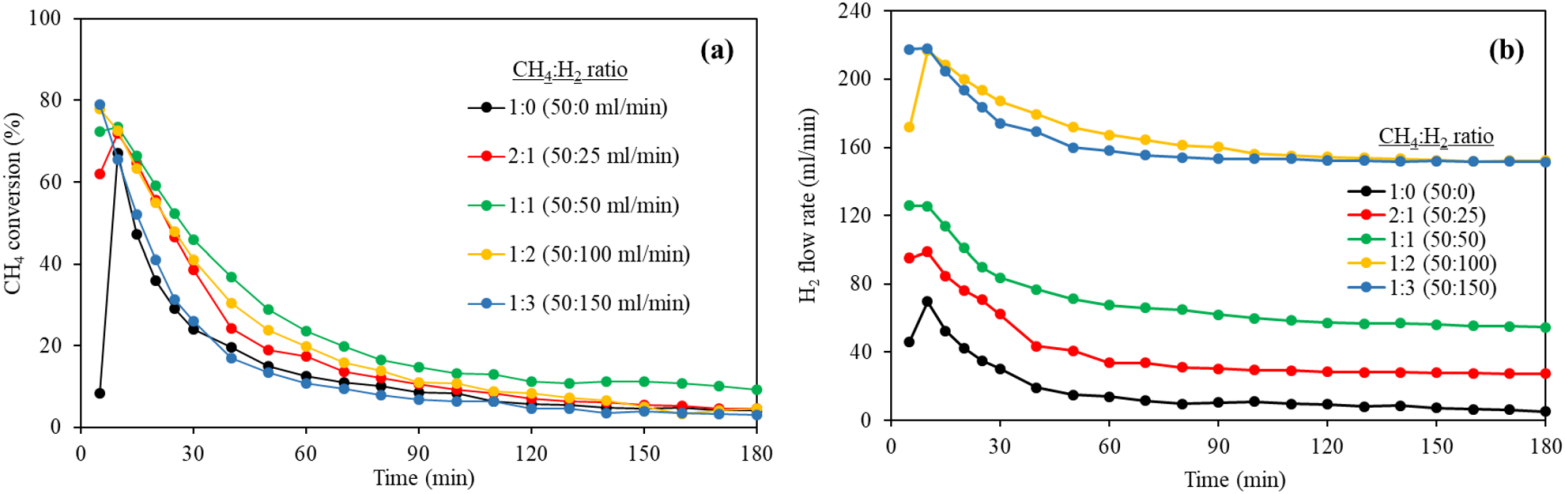
Effect of H_2_ concentration on (**a**) CH_4_ conversion (**b**) calculated H_2_ flow rate over FeMo/MgO catalyst at 900 °C.

**Figure 12:**
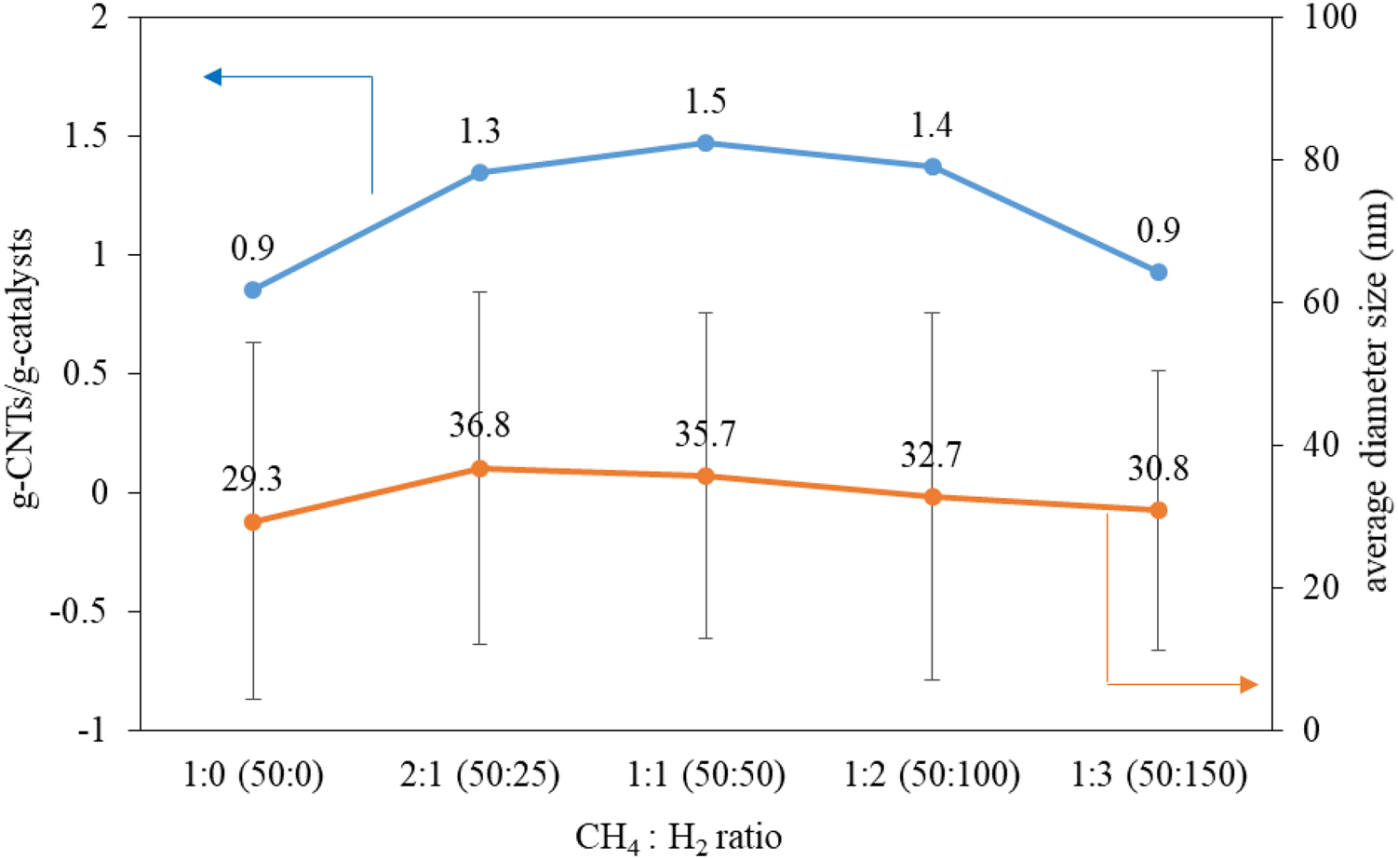
g-CNTs/g-catalyst, and average CNTs diameter as a function of CH_4_:H_2_ flow rate ratio. Error bars are the standard deviation for each sample and is a measure of the diameter distribution.

Effects of H_2_ concentration were studied with H_2_ co-feed or nR-wH_2_ process. The catalytic performance was investigated at varied CH_4_:H_2_ ratio which are 1:0 (50:0 ml min^−1^, nR-woH_2_), 2:1 (50:25 ml min^−1^), 1:1 (50:50 ml min^−1^), 1:2 (50:100 ml min^−1^, nR-wH_2_), and 1:3 (50:150 ml min^−1^). N_2_ was used to adjust total flow rate to 200 ml min^−1^. Methane conversion was investigated and H_2_ flow rate was calculated by Eq. 4 then plotted against reaction time as shown in Fig. 11. As-prepared CNTs were measured for average diameter and summarizing the results with other information in Fig. 12 and Table 4.

**Table 4:** The summary data for study of effects of H_2_ concentration.

CH_4_: H_2_ ratio	Product weight	%Yield	g-CNTs/g-catalyst	D_avg_ (nm)	Sd. (nm)
1:0 (50:0 ml min^−1^)	0.9294	10.16	0.85	29.28	25.03
2:1 (50:25 ml min^−1^)	1.1763	17.06	1.35	36.81	24.72
1:1 (50:50 ml min^−1^)	1.2401	17.06	1.47	35.72	22.82
1:2 (50:100 ml min^−1^)	1.1889	15.85	1.37	32.72	25.80
1:3 (50:150 ml min^−1^)	0.9669	10.51	0.93	30.81	19.64

Methane conversion in each H_2_ concentration was slightly different as shown in Fig. 11a, where the CH_4_:H_2_ ratio was 1:1 with the highest methane conversion. In Fig. 11b, exhibited H_2_ flow rate that is higher than amount of H_2_ fed into the system. Since H_2_ is one of the products resulting from the decomposition of methane, it was found that H_2_ flow rate decreases over time corresponding to the reduced methane conversion.

Figure 12 shows the yield of the produce (g-CNTs/g-catalyst) with an optimum point of 1:1, corresponding to the conversion of methane. When the H_2_ content is increased above 1:1, the yield is less. Observing the flow rate of H_2_, found that there is high amount of H_2_ in system. The study of Kashiwaya Y., and Watanabe M. reported that methane decomposition is reversible reaction in gas phase before CNTs growth^42^. Therefore, according to Le Chatelier’s principle, when the product quantity (H_2_) increases, will prevent the dissociation of methane, resulting in fewer CNTs^43,44^. Additionally, the addition of the proper amount of H_2_ allows catalyst to restructure that ready for the formation of higher CNTs. Observed from H_2_-free process (nR-woH_2_) with the lowest methane conversion (~ 8%) at 5 min, then the conversion increased. Because initially the catalyst was in an oxide form with lower methane conversion performance, and when partially H_2_ was formed, the catalyst changed its structure and thus the conversion increased.

The results of the study on adjustment concentration of H_2_ gas, when adding H_2_, yield was higher but decreased when excess H_2_. Therefore, adding H_2_ to CNTs product has an optimal point, that consistent with various research^12–18^.

Measure and establish the CNTs diameter size distribution from the study of effects of H_2_ concentration as shown in Fig. 12 and Table 4, found that as-prepared CNTs from this work are highly distributed. As a result, it was unable to confirm the results of the study of the diameter changes from the adjustment of the H_2_ concentration.

However, several studies indicate that H_2_ addition helps to eliminate amorphous carbon as well as maintain catalyst stability. To study about this issue, the study of catalytic hydrogenation reaction was conducted in the next section.

#### Hydrogenation reaction

This section examined the catalytic hydrogenation reaction that was thought to play an important role in the synthesis of CNTs, by investigating whether this reaction can help stabilize the catalyst during the synthesis of CNTs, included with an improvement of CNTs quality.

Piedigrosso et al.^11^ reported that, CNTs are capable of catalytic hydrogenation, but there is no selective eliminate of solid carbon, either crystalline carbon or amorphous carbon. Catalytic hydrogenation may enhance the catalyst performance by removing the carbon covering the catalyst surface, which caused the deactivation of catalyst. In this work, an experiment was conducted to study the synthesis of CNTs in reduced-process, by comparing between processes with and without H_2_ feed (R-woH_2_ and R-wH_2_) with a reaction time of 5 h (300 min) to study catalyst stability of both processes (Figure S2). It was found that the conversion of methane continues to decrease at the same rate in both processes. Comparison the product weight difference between two processes, at 3 h, the difference was about 0.07 g, but at 5 h, the difference was about 0.26 g. Therefore, it seems that addition of H_2_ does not keep catalyst stability, but also reduces the weight of the product.

The catalytic hydrogenation reaction was tested to prove these issues. As-prepared CNTs from R-woH_2_ and R-wH_2_ process were tested for catalytic hydrogenation reaction at 900 °C with flow rates of 100 and 50 ml min^−1^ of H_2_ and N_2_, respectively. Effluent gas, product weight, and crystalline ratio were analyzed to identify the behavior that occurred.

**Figure 13:**
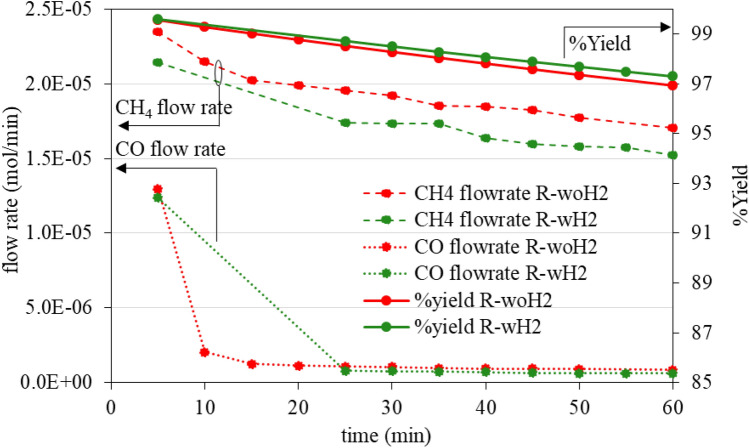
Flow rate of CH_4_ and CO, and %yield as a funtion of catalytic hydrogenation reaction time.

Figure 13 exhibits flow rate of CH_4_ and CO released from the system during the catalytic hydrogenation reaction test. It also shows the calculated quantity of CNTs consumed as a %yield. From the experiment, it was found that CO was initially formed. Methane occurred throughout the experiment, with the flow rate decreasing slightly over time. After the experiment, approximately 3% of CNTs were consumed. In addition, CH_4_ and CO generated rate have no different for each process, although these two samples are expected to have different incidences, because R-wH_2_ has been occurred in catalytic hydrogenation (during the formation of CNTs) for longer than R-woH_2_. If catalytic hydrogenation is a selective reaction, a difference will occur in some way.

**Figure 14:**
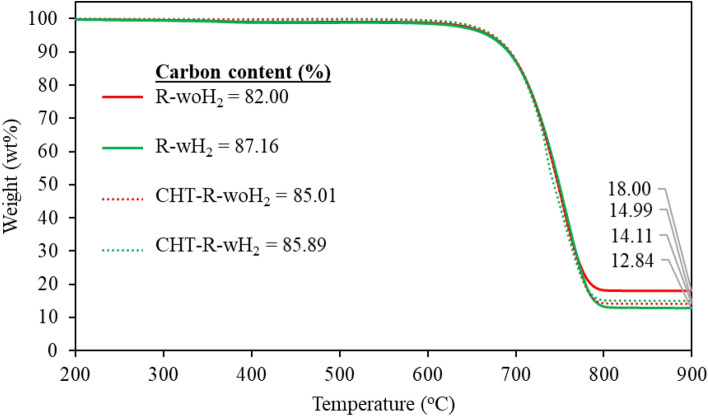
TGA of CNTs for study of catalytic hydrogenation reaction.

The thermal stability of the product comparing between As-received CNTs (from process R-woH_2_ and R-wH_2_) and CNTs after process Catalytic hydrogenation reaction (CHT) is shown in Fig. 14, and the average crystallinity ratio by Raman characteristic is shown in Table 5.

**Table 5:** Purity and I_G_/I_D_ ratio for study of catalytic hydrogenation reaction.

Experimental	%purity (TGA)	Avg. I_G_/I_D_
R-woH_2_	82.00	2.58 ± 0.49
R-wH_2_	87.16	2.56 ± 0.24
CHT-R-woH_2_	85.01	2.61 ± 0.45
CHT-R-wH_2_	85.89	2.60 ± 0.14

From the thermal decomposition in Fig. 14, it was found that all samples were not different, with similar carbon content and decomposed at the same temperature range. Consider the proportion of crystallinity. Raman analysis has shown no difference in I_G_/I_D_ ratio with an average of 2.6.

Therefore, from the study of the catalytic hydrogenation reaction. It can be concluded that the catalytic hydrogenation reaction occurs during the synthesis of CNTs, whereas CNTs are used as a precursor to form methane and carbon monoxide. This reaction was not selective to eliminate either crystalline or amorphous carbon. As a result, it does not improve the stability of the catalyst, by etching carbon from surface of catalyst. The addition of H_2_ to the system will not enhance the properties of CNTs and produce less product, because of the catalytic hydrogenation reaction and the shifted of reaction equilibrium.

From the study of the role of H_2_ in “Effect of the presence of H2” section, it was concluded that an important role of H_2_ is to determine the catalyst structure by H_2_ pre-reduction of catalyst before CNTs formation. Whereas the addition of H_2_ during the formation of CNTs was not found to have a beneficial effect on the synthesis of CNTs and resulted in less product yield.

### Kinetic study of CNTs synthesis

**Figure 15:**
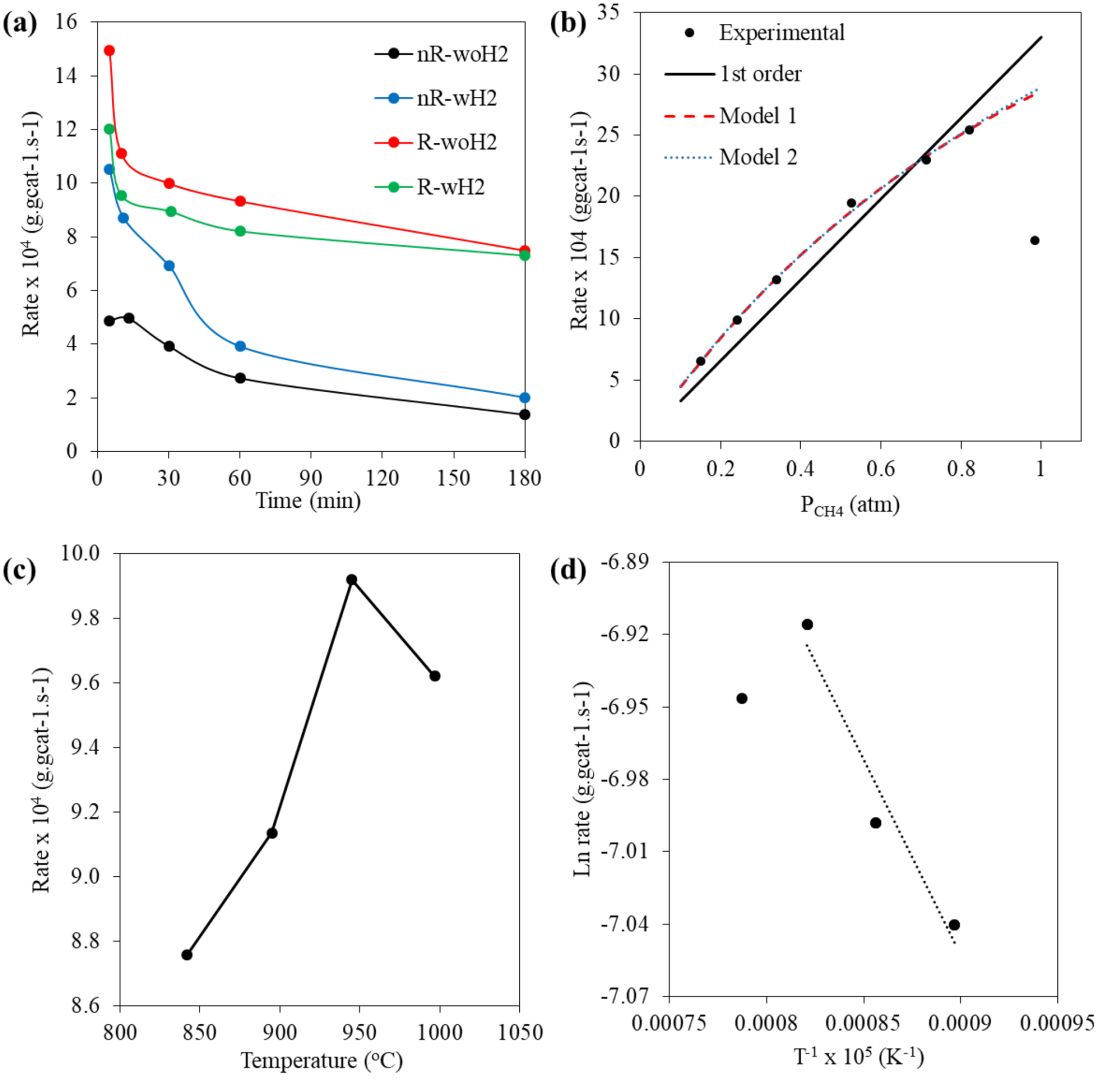
(**a**) The yield rate of CNTs synthesis at 900^o^C, (**b**) Effect of partial pressure of methane on the yield rate of CNTs formation at 900^o^C with line indicating the calculated rates by kinetic model, (**c**) Effect of temperature on the CNTs yield rate, and (**d**) The Arrhenius plot for estimation of the activation energy.

The aim of the kinetic study of CNTs synthesis was to investigate the mechanisms occurring during the synthesis of CNTs, with the hope that the data could be used for further process scale-up. Data collection for use in kinetic studies in CNTs synthesis is quite difficult because solids form while reaction that made an unsteady state reaction. Research works have chosen a variety methods to investigated reaction rate, such as measuring the height of VACNTs^45^, analysis of hydrogen output gas^46^, or batch analysis by collecting data on changing weight of the catalyst bed^47,48^. In this work, batch analysis was used. The reaction rate in each process was collected as shown in Fig. 15a. The rate of product yield was calculated according to the Eq. (6), with units of gram product divided by grams of catalyst and reaction time in second. A dramatic decrease in yield rate can be observed in all processes. From the study in “Roles of hydrogen” section it was found that R-woH_2_ process had the highest yield and was able to reduce the complexity of analysis by removing H_2_ feeding. Therefore, this process was chosen for further kinetic studies with 30 min reaction time. The partial pressure of CH_4_ in feed gas was varied to identify the reaction order and effects of CH_4_ concentration. The experimental data for kinetic study was shown in Table S1. Partial pressure of CH_4_ was conducted by varying of CH_4_ flow rate.

Yadav et al.^49^ derived model 1 and 2 by assuming dissociative adsorption of methane followed by removal of hydrogen from the adsorbed methyl group as the rate determining step. The reaction step was considered as irreversible reaction and the difference between model 1 and 2 is the one type of active site (x) or two types (x and y). Pseudo first order reaction kinetic, kinetic model 1 and model 2 were used to compare for this reaction, leading to the following rate Eqs. (7–9). The rate constants are given by k^’^, k_1_ and k_2_, respectively. To estimate the parameters k, Eqs. (7–9) can be linearized and parameters were estimated from the slope and intercepts.

7 $${1}^{{{\text{st}}}} \;{\text{order}}\;{\text{reaction}}\;{\text{kinetic}}\;R = k^{\prime } P_{{CH_{4} }}$$

8 $${\text{Model}}\;{1}\;R = \frac{{k_{1} P_{{CH_{4} }} }}{{1 + \frac{{k_{1} P_{{CH_{4} }} }}{{k_{2} }}}}$$

9 $${\text{Model}}\;{2}\;R = \frac{{k_{1} P_{{CH_{4} }} }}{{1 + \frac{{k_{1} P_{{CH_{4} }} }}{{k_{2} }}^{2} }}$$

The experimental and the simulated values from models were plotted as shown in Fig. 15b. The partial pressure of methane equal to 1 does not consider as it is a pure methane system which is inconsistent with the other proportions. The rate of formation of CNTs increased with an increase in the partial pressure of methane, then reaches as saturation level followed by decrease in the rate at higher partial pressures of methane. The value of the rate constant from each model was shown in Table 6. Yadav et al. resulted as well. It can be seen that model 1 predicts the reaction rate same as model 2 which is better than 1st order reaction over a covered condition in this work. These results show that the decomposition of methane into CNTs and hydrogen is might be 1st order reaction but, model 1 and model 2 can predict more accurately.

**Table 6:** Estimated values of the kinetic parameters of Model 1, Model 2 and 1st order reaction.

Reference	Temperature (^o^C)	Model 1	k_2_ × 10^4^ ${\text{(g g}}_{{{\text{cat}}}}^{{ - 1}} {\text{ s}}^{{ - 1}} )$	R^2^	Model 2	k_2_ × 10^4^ $({\text{g}}\;{\text{g}}_{{{\text{cat}}}}^{ - 1} \;{\text{s}}^{ - 1} )$	R^2^	1st order reaction	R^2^
		k_1_ × 10^3^ ${\text{(g}}\;{\text{g}}_{{{\text{cat}}}}^{{ - {1}}} \;{\text{s}}^{{ - {1}}} \;{\text{atm}}^{{ - {1}}} {)}$			k_1_ × 10^3^ $({\text{g}}\;{\text{g}}_{{{\text{cat}}}}^{ - 1} \;{\text{s}}^{ - 1} \;{\text{atm}}^{ - 1} )$			k × 10 $({\text{g}}\;{\text{g}}_{{{\text{cat}}}}^{ - 1} \;{\text{s}}^{ - 1} \;{\text{atm}}^{ - 1} )$	
Present work	900	4.81	72.31	0.998	4.74	165.28	0.998	3.30	0.938
Yadav et al.^49^	850	6.26	2.38	0.978	3.98	8.84	0.996	-	-
	950	14.4	2.38	0.982	5.85	9.98	0.996	-	-

The effect of temperature was studied in the range of 850–1000 °C with partial pressure of methane equal to 0.24 atm. (50 ml min^−1^). It was observed that the rate of CNTs formation increased with temperature until higher than 950 °C because of catalyst begin to be agglomerated that decreased catalyst performance, as shown in Fig. 15c.

To estimate the activation energy (E_a_), Arrhenius plot is needed. Then the rate of reaction (lnR_CNTs_) was plotted versus T^−1^ as shown in Fig. 15d. The rate of CNTs formation at 1000 °C was not considered for E_a_ estimation, as agglomeration of active phase occurred.

The activation energy was found to be 13.22 kJ mol^−1^, compared to the other tasks in summarize table of Yadav et al.^49^ work, our E_a_ was much lower. It may be in a range of mass transfer limit^50^, consistent with an assumption of pseudo first order reaction that used to describe mass transfer limit the reaction rate. Additionally, our experiment found the behavior of the CNTs formation rapidly occurs, and a compaction of the bed layer will occur, which may hinder the formation of CNTs, showing that this experimental process undesignated for study mass transfer limit process. Therefore, it is possible that E_a_ has a lower value compared to other works. Moreover, E_a_ determinations should be carried out in reactions with low conversion and reaction temperatures to get accurate and reasonable values ^51,52^.

## Conclusion

In this study, the role of H_2_ on CNTs synthesis was investigated by means of pre-reduction of catalyst and co-feeding during CNTs synthesis. For FeMo/MgO catalyst, the reduced process could produce CNTs with the largest yield ascribed to the presence of highly active FeMo metallic phases. Meanwhile, the Mo_2_C phase was formed in the non-reduced process, resulting in the formation of CNTs with smaller diameter and higher crystallinity. The co-feeding of H_2_ during CNTs synthesis in the non-reduced process can increase the yield of CNTs formation. However, the yield became lower when H_2_ exceeded the equilibrium of reaction. In addition, the presence of H_2_ during the CNTs synthesis would promote the catalytic hydrogenation of carbon to methane. This reaction was not selective for elimination of amorphous carbon. As a result, the co-feeding of H_2_ would not improve neither the CNTs crystallinity nor the stability of the catalyst. The kinetic study of CNTs synthesis shows that methane decomposition to CNTs and hydrogen is a pseudo first order reaction with mass transfer as rate-controlling step. The rate of CNTs formation increases with an increased methane partial pressure. The activation energy derived from the experimental data was found to be relatively low at 13.22 kJ mol^−1^. In sum, the synthesis of MWCNTs by CCVD of methane using FeMo/MgO catalyst can be achieved under H_2_ free condition. The characteristics of as-grown CNTs would greatly depend on the structure of the catalyst. The adding of hydrogen shows an important role in changing the catalyst structure during the pre-reduction of catalyst.

### Supplementary Information

Supplementary Information.

## Data Availability

All data related to the finding of this study are accessible upon request from the corresponding author Apinan Soottitantawat.
