# Supplementary material for: Synthesis of MWCNTs by chemical vapor deposition of methane using FeMo/MgO catalyst: role of hydrogen and kinetic study
Source: Sci Rep. 2023 Nov 29;13:21027. doi: 10.1038/s41598-023-48456-z (PMC10687016; doi:10.1038/s41598-023-48456-z)

Supplementary - **Synthesis of MWCNTs by Chemical Vapor Deposition of Methane Using FeMo/MgO catalyst: Role of Hydrogen and Kinetic Study**


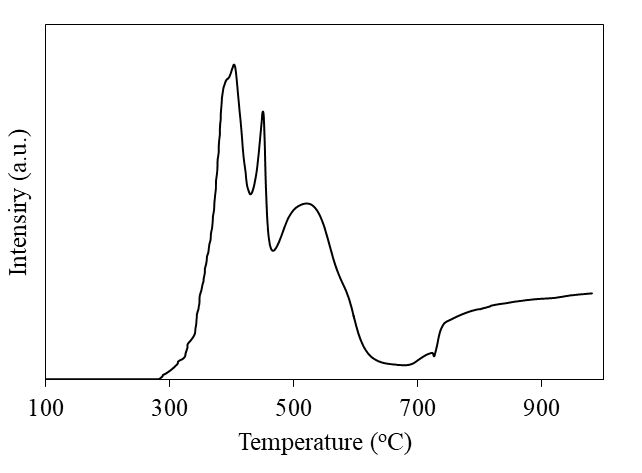


Figure S1 H_2_-TPR analysis of FeMo/MgO catalyst


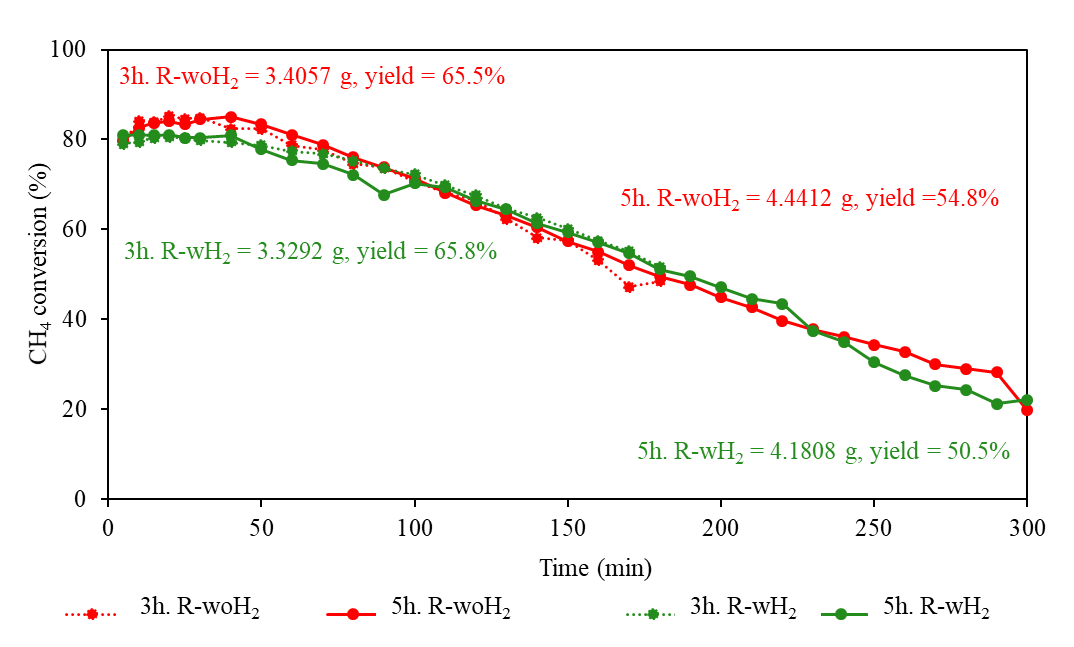


**Figure S2** CH_4_ conversion over FeMo/MgO catalyst between R-woH_2_ and R-wH_2_ with reaction time 3h. (180 min) and 5h. (300 min)

**Table S1** Experimental data for kinetic study of CNTs formation


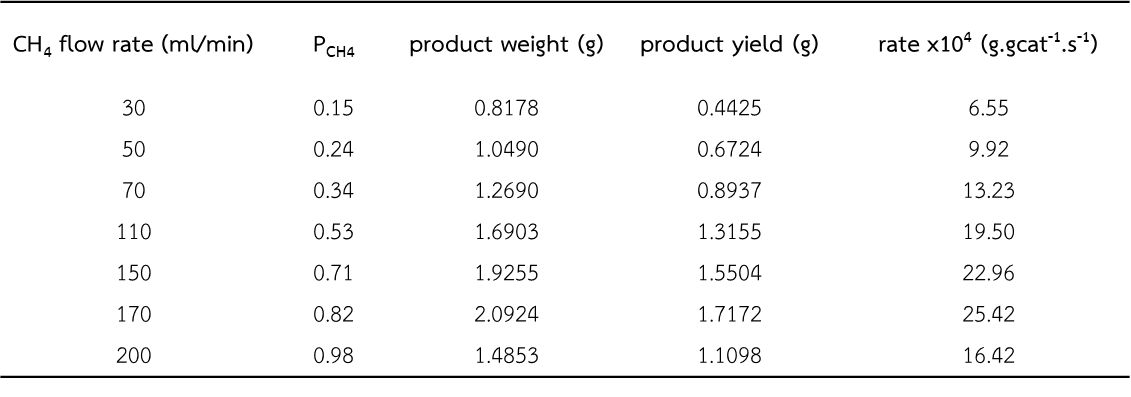

Supplement: Supplementary file 1 — Supplementary Information. [file 41598_2023_48456_MOESM1_ESM.docx]
